# Implementation of laparoscopic hysterectomy for endometrial cancer over the past decade

**DOI:** 10.1186/s10397-018-1040-x

**Published:** 2018-02-27

**Authors:** Tim Wollinga, Nicole P. M. Ezendam, Florine A. Eggink, Marieke Smink, Dennis van Hamont, Brenda Pijlman, Erik Boss, Elisabeth J. Robbe, Huy Ngo, Dorry Boll, Constantijne H. Mom, Maaike A. van der Aa, Roy F. L. P. Kruitwagen, Hans W. Nijman, Johanna M. A. Pijnenborg

**Affiliations:** 1 Medical Faculty, Erasmus University, Rotterdam, The Netherlands; 2 Department of Obstetrics and Gynecology, Elisabeth-Tweesteden Hospital, Tilburg, The Netherlands; 3 Department of Medical and Clinical Psychology, Tilburg University, Tilburg, The Netherlands; 4 Netherlands Comprehensive Cancer Organisation, Utrecht, The Netherlands; 5 Department of Obstetrics and Gynecology, University Medical Centre Groningen, University of Groningen, Groningen, The Netherlands; 6 Department of Obstetrics and Gynecology, Amphia Hospital, Breda, The Netherlands; 7 Department of Obstetrics and Gynecology, Jeroen Bosch Hospital, ‘s-Hertogenbosch, The Netherlands; 8 Department of Obstetrics and Gynecology, Máxima Medical Centre, Veldhoven, The Netherlands; 9 Department of Obstetrics and Gynecology, St. Anna Hospital, Geldrop, The Netherlands; 10 Department of Obstetrics and Gynecology, Elkerliek Hospital, Helmond, The Netherlands; 11 Department of Obstetrics and Gynecology, Catharina Hospital, Eindhoven, The Netherlands; 12 Department of Gynecologic Oncology, Gynecological Oncology Centre Amsterdam, Amsterdam, The Netherlands; 13 Department of Research, Netherlands Comprehensive Cancer Organisation, Utrecht, The Netherlands; 14 Department of Obstetrics and Gynecology, Maastricht University Medical Centre, Maastricht, The Netherlands; 15 GROW-School for Oncology and Developmental Biology, Maastricht University, Maastricht, The Netherlands; 16 Department of Obstetrics and Gynecology, Radboud University Medical Centre, 791, P.O. Box 9101, 6500 HB Nijmegen, The Netherlands

**Keywords:** Endometrial cancer, Laparoscopic hysterectomy, Implementation

## Abstract

**Background:**

Laparoscopic hysterectomy (LH) for the treatment of early-stage endometrial carcinoma/cancer (EC) has demonstrated to be safe in several randomized controlled trials. Yet, data on implementation of LH in clinical practice are limited. In the present study, implementation of LH for EC was evaluated in a large oncology network in the Netherlands.

**Results:**

Retrospectively, a total of 556 EC patients with FIGO stage I-II were registered in the selected years. The proportion of LH gradually increased from 11% in 2006 to 85% in 2015. LH was more often performed in patients with low-grade EC and was not related to the studied patient characteristics. The introduction of TLH was frequently preceded by LAVH. Patients treated in teaching hospitals were more likely to undergo a LH compared to patients in non-teaching hospitals. The conversion rate was 7.7%, and the overall complication rates between LH and AH were comparable, but less postoperative complications in LH.

**Conclusions:**

Implementation of laparoscopic hysterectomy for early-stage EC increased from 11 to 85% in 10 years. Implementation of TLH was often preceded by LAVH and was faster in teaching hospitals.

## Background

Endometrial carcinoma/cancer (EC) is the most common malignancy of the female genital tract, with an increasing incidence in western countries [1]. In the Netherlands, about 1900 women are diagnosed with EC yearly. Due to the fact that most patients are diagnosed at an early stage, outcome is relatively favorable. However, 400 women die of this disease annually in the Netherlands [2]. Primary treatment consists of a total hysterectomy and bilateral salpingo-oophorectomy. Routine lymphadenectomy is not beneficial in early-stage, low-risk EC [3]. Historically, hysterectomy for EC was performed by laparotomy. In 1989, Harry Reich performed the first laparoscopic hysterectomy (LH) for a benign gynecological disease [4]. Subsequently, LH became an alternative approach for abdominal surgery with the advantage of an increased recovery time and reduced blood loss [5]. In 2006, the results from the Laparoscopic Approach to Carcinoma of the Endometrium (LACE) trial, a randomized controlled trial (RCT) including 509 patients with stage I EC, were published. This study demonstrated a similar survival in patients treated by total laparoscopic hysterectomy (TLH) compared to those who underwent abdominal hysterectomy (AH). The benefits of a laparoscopic approach included shorter hospital stay, less analgesics, and reduced perioperative morbidity [6]. These data were confirmed by a Dutch RCT including 283 patients with stage I EC, who were also randomized between TLH and AH [7]. The benefits of a laparoscopic approach seem to be even more relevant in obese patients with an increased risk of surgical morbidity [8]. This is particularly relevant in EC, as obesity is an important risk factor for the development of EC [9]. Yet, especially in morbidly obese patients, laparoscopic surgery for EC requires a well-trained team, with a completed learning curve of the surgeon, and anesthesiologists that are used to steep Trendelenburg position during surgery [10]. Over the last decades, the percentage of LH as a primary surgical treatment in EC has increased [11]. Factors that might influence the adoption of LH in clinical practice have been reported to be related to age and sex of the gynecologist and the presence of gynecology residents [12–14].

Previous studies have focused on the implementation of laparoscopy in general gynecology in the Netherlands and have shown a slowly increase of LH from 3% in 2007 to 10% in 2012 [15]. However, these data are based on questionnaires sent to gynecologists about their performed surgeries. So far, only a few studies have focused on the implementation of LH in the surgical treatment of EC. In a recent study, out of 5239 hysterectomies for EC in the USA, 51% was performed by laparoscopy in 2012 [16]. Bogani et al. demonstrated in a single-center study the implementation of laparoscopic surgery in the management of all types of gynecological cancers and showed an increase from 10 to 82% in a 10-year time period [17]. The aim of the present study was to evaluate the implementation of LH in the treatment of early-stage EC over the past 10 years in a large clinical oncology network and to determine which patient-, hospital-, and surgeon-related factors contributed to the implementation of LH. In addition, we evaluated the conversion and complication rates as well as the duration of laparoscopic surgery in relation to the annual number of EC surgeries per hospital.

## Methods

### Setting

A retrospective cohort study was performed in the Gynecological Oncology Centre South (GOCS), a clinical oncology network in the south of the Netherlands. The GOCS comprises eight collaborating hospitals: two oncological referral centers, four teaching hospitals, and two non-teaching hospitals. According to Dutch guidelines, surgical staging and lymph node dissection in clinical stage I, endometrioid-type EC are recommended only in case of clinical suspicion of lymph node metastasis or in case of high-grade histology, i.e., grade 3 endometrioid-type and all non-endometrioid-type EC cases. Adjuvant therapy consists of radiotherapy by either external beam radiation or vaginal vault brachytherapy, depending on the patient’s age, myometrial invasion, LVSI, and tumor grade on final pathology [18, 19].

### Patients

All patients that underwent primary surgical treatment for EC in 2006, 2009, 2012, or 2015 within the GOCS were included. Patients planned for a hysterectomy for another reason (*n* = 13), e.g., uterine myomas, but were diagnosed postoperatively with EC, were documented, but were excluded for analysis since the planned surgery was not based on the preoperative diagnosis of EC. Patients that received neoadjuvant chemotherapy, radiotherapy, or hormonal therapy were excluded (*n* = 5), as well as patients that were diagnosed with other uterine tumors (*n* = 10). Patients were classified according to the 2009 International Federation of Gynecology and Obstetrics (FIGO) staging system [20, 21]. Routine preoperative work-up in the Netherlands consists of a chest X-ray for low-risk (grade 1–2) endometrioid-type EC, and computed tomography scan for high-risk tumors, and only in low-grade EC when there is clinical suspicion of extended disease. Determination of myometrial invasion is not routinely performed (www.oncoline.nl).

### Data extraction

Patient and tumor characteristics were extracted from patient files, pathology reports, and anesthesiological screenings. The following patient characteristics were collected: body mass index (BMI), comorbidity, previous surgery, and smoking habit. Both the planned and the performed surgical approaches were registered. LH was categorized into TLH and laparoscopic-assisted vaginal hysterectomy (LAVH). TLH was defined as a complete laparoscopic surgical approach including closure of the vaginal vault. When part of the procedure was done vaginally, including closure of the vaginal vault, it was recorded as LAVH. For patients that were planned for a laparoscopic approach but underwent AH, the reason for conversion was documented according to the following factors: adhesions, limited exposure, anesthesiological difficulties due to Trendelenburg position, and uncontrolled bleeding. In addition, complications during surgery and postoperatively were documented.

### Outcome

Primary outcome was defined as the percentage of laparoscopic hysterectomies in all patients with FIGO stage I–II EC in 2006, 2009, 2012, and 2015, compared to the percentage of abdominal hysterectomies. Secondary outcomes were the determination of predictive factors for a laparoscopic approach and the relation between annual surgical volume and duration of surgery. Predictive factors for the laparoscopic approach were classified as patient, hospital, and surgeon related (age and gender). The type of hospital was classified as teaching and non-teaching hospital. Surgeon-related factors were age and gender of the surgeon. The duration of the surgical procedures (LH and AH) was related to the annual number of patients undergoing surgery for EC within the hospitals.

### Statistical analysis

Descriptive analyses were used to describe patients treated with LH and AH. Differences in characteristics between patients treated with LH and AH were assessed using Student’s *t* tests for continuous variables and the chi-square test for categorical data. A multivariable logistic regression was performed to determine the association of the following patient-, hospital-, and surgeon-related factors with the likelihood of a laparoscopic hysterectomy: age of the patient, previous abdominal surgery, BMI, and diabetes mellitus. Patients who could potentially receive either LH or AH were included, i.e., those treated in 2012 and 2015, since LH was implemented in most hospitals in these years and those patients with a grade I and stage I and II endometrial cancer [11]. Statistical analyses were conducted using IBM SPSS Statistics, version 20 (SPSS Inc., Chicago, IL, USA). *p* values < 0.05 were considered statistically significant, and all statistical tests were two-sided.

## Results

### Patient cohort

**Fig. 1:**
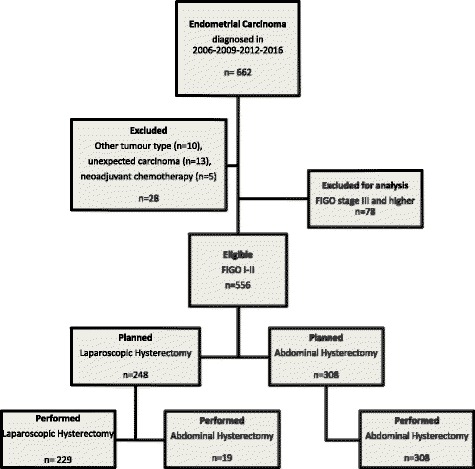
Flow chart of the patient inclusion and surgical procedures

A total of 662 patients were diagnosed with EC within the selected years: 2006, 2009, 2012, and 2015. Twenty-eight patients were excluded due to other tumor types (*n* = 10), neoadjuvant chemotherapy (*n* = 5), and unexpected EC (*n* = 13). Subsequently, for analysis, only patients with FIGO stage I–II EC were included, resulting in 556 eligible patients, as demonstrated in Fig. 1. Patient and tumor characteristics are demonstrated in Table 1. There were no differences between patients that underwent a LH or AH with respect to age, BMI, parity, smoking, or duration of surgery. There were 19 conversions to laparotomy (7.7%): 14 due to adhesions, two due to uncontrolled bleeding, two due to anesthesiological difficulties, and one due to limited exposure. The conversions were different over time: 2006 (25.0%), 2009 (3.3%), 2012 (10.0%), and 2015 (6.9%). Previous abdominal surgery was not a risk factor for conversion to laparotomy (*p* = 0.722). Although there was no difference in the overall complication rate between LH and AH, perioperative complications were observed more frequently in the LH group, whereas postoperative complications were observed more frequently in the AH group. Complications during surgery in the AH group consisted of the following: intestinal wall injury (*n* = 3), bleeding (*n* = 3), and damage to the obturator nerve (*n* = 1). Complications in the LH group consisted of the following: intestinal wall injury (*n* = 10), bleeding (*n* = 4), injury to the bladder (*n* = 2), rupture of the cervix (*n* = 2), and uterine rupture (*n* = 1). Postoperative complications in the AH group consisted of wound dehiscence (*n* = 18), urinary tract infection (*n* = 3), wound infection (*n* = 2), and postoperative bleeding (*n* = 1). Complications after surgery in the LH group consisted of wound infection (*n* = 4), urinary tract infection (*n* = 1), and wound dehiscence (*n* = 1).

**Table 1:** Patient and tumor characteristics in relation to the type of performed surgical procedure

Treatment characteristics of all included patients	Total, *n* = 556	LH, *n* = 248	AH, *n* = 308	*p* value
Age in years (mean, SD)	65.8 (9.6)	65.3 (9.8)	66.4 (9.4)	0.166
BMI (mean, SD)	29.6 (6.7)	29.8 (7.0)	29.3 (6.3)	0.429
Parity (mean, SD)	2.0 (1.4)	2.03 (1.4)	2.02 (1.3)	0.909
Previous abdominal surgery	184	80	104	0.585
Comorbidity				
Hypertension	178	77	101	0.582
Type II diabetes	83	30	53	0.093
FIGO stage				0.364
IA	336	158	178	
IB	195	80	115	
II	25	10	15	
Histology				0.002
Endometrioid	502	231	271	
Non-endometrioid				
Serous	28	15	13	
Clear cell	8	2	6	
Carcinosarcoma	15	0	15	
Stromal cell sarcoma	3	0	3	
Tumor grade				0.000
1	279	151	128	
2	175	63	112	
3	102	34	68	
Treatment				
Hysterectomy and BSO	556	305	241	0.196
Additional staging/lymphadenectomy	80	10	70	0.000
Conversion to laparotomy	19	19	–	
Duration of surgery, min (mean, SD)	101 (41.9)	116 (39.3)	90 (40.4)	0.204
Complications				
During surgery	26	19	7	0.004
After surgery	30	6	24	0.005

*LH* laparoscopic hysterectomy, *AH* abdominal hysterectomy

### Surgical procedure during the years

**Fig. 2:**
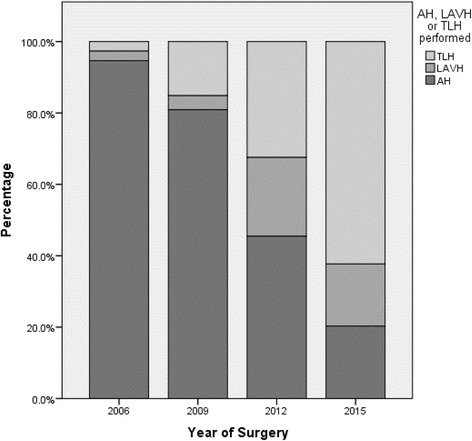
Description of the proportion of surgical procedures (TLH, LAVH, or AH) in the years 2006, 2009, 2012, and 2015

During the study period, a steady rise of both LAVH and TLH was demonstrated, as illustrated in Fig. 2. In 2006, only eight (11%) patients underwent surgery by the laparoscopic approach, compared to 30 (19.7%) in 2009, 93 (60%) in 2012, and 117 (85%) in 2015. One teaching hospital introduced the laparoscopic approach for the treatment of EC in 2006. The remaining teaching hospitals started performing laparoscopic surgery between 2006 and 2015. The two non-teaching hospitals initiated LH for EC between 2012 and 2015. In five hospitals, the introduction of LAVH preceded the implementation of TLH.

### Predictors of laparoscopic hysterectomy

There was no relation between patient-related factors, such as BMI (OR 1.00, 95% CI 0.98–1.0), previous abdominal surgery (OR 1.06, 95% CI 0.89–1.26), age (OR 0.98, 95% CI 0.97–1.00), type II DM (OR 0.59, 95% CI 0.34–1.02), and hypertension (OR 1.00, 95% CI 0.66–1.50) with the type of surgical approach. In addition, there was no relation between surgeon-related factors and type of surgery. Patients that underwent surgery in teaching hospitals were more likely to be operated by a laparoscopic approach compared to non-teaching hospitals (OR 4.65, 95% CI 2.59–8.36).

### Duration of surgery

**Fig. 3:**
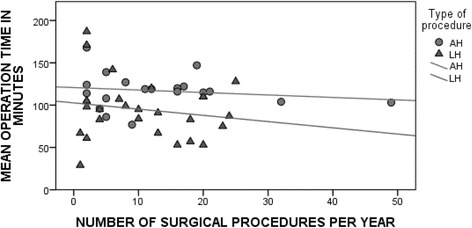
Mean duration of surgery in relation to the annual number of operated endometrial cancer patients

For all hospitals, the mean duration of surgery was calculated for LH and AH and related to the number of EC patients operated annually. As illustrated in Fig. 3, the mean duration of AH was independent of the number of performed procedures. Yet, for the duration of LH, there was a trend towards a longer operating time when less EC patients were treated per year.

## Discussion

This study showed an imposing increase in laparoscopic treatment of early-stage EC from 11% of the procedures in 2006 to 85% in 2015, reflecting that LH was well implemented in the past decade in the studied clinical oncology network in the Netherlands. The introduction of TLH was frequently preceded by LAVH. The only predictive factor for a laparoscopic approach was treatment in a teaching hospital.

To the best of our knowledge, this is the first study that reports upon the implementation of LH in the treatment of EC over a 10-year period since the publication of the LACE trial in 2006. In a recently published study, results over a 4-year time span demonstrated an increase in minimally invasive hysterectomy of 22% in 2007 to 51% in 2011 in the USA [22]. Data are in line with results from Bogani et al. who compared the type of surgical approach for gynecological malignancies during the years 2000–2003 with 2008–2011 and showed a comparable increase from 10 to 82%. Yet, these data were from a single center and included large numbers that might explain a faster increase in implementation [17]. In comparison, the implementation of LH in the Netherlands was relatively late when compared to that in other countries, possibly due to the lack of centralization of EC treatment resulting in many hospitals treating small numbers [23]. Implementation in the Netherlands might have been facilitated by the Dutch RCT, published in 2010 [7].

The observed conversion rate changed over time and was 6.9% in the last year of our study, quite in line with the previous Dutch RCT that reported conversion rates of 10.8%, but higher than the reported 2.4% in the LACE trial [6, 7]. Even in 2015, this number is still relatively high. Possible explanations are as follows: (1) variations in the time of the start of LH between hospitals that may not have reached the optimal surgical performance at the time of analysis, (2) relatively small numbers per hospital, and (3) a substantial proportion of obese patients (40.6%), since these are associated with increased conversion rate [23, 24]. The overall comparable complication rates support our assumption that laparoscopic surgeons in the GOCS region were sufficiently trained to perform a LH. The absence of a decrease in the rate of complications with the implementation of LH during the 10-year period can be explained by the fact that more surgeons started to perform LH for endometrial cancer, each going through their individual learning curve. Analyses of an *overall* learning curve are thus a mixture of several *individual* learning curves. The observed trend towards an increased duration of surgery with less LH cases per year suggests that surgical volume might be relevant. However, since surgeons that perform LH of endometrial cancer also perform LH for benign indications, these numbers should be included for a proper analysis. The observation that the introduction of a TLH was frequently preceded by a LAVH approach may illustrate a step-wise adaptation of laparoscopic surgery. Although we hypothesized, according to previous findings, that patient-related factors such as BMI and previous abdominal surgery were predictive for the type of surgical approach, we could not confirm this in our study [24, 25]. In our study cohort, 72.6% of the patients were overweight, with 40.6% being obese. The Dutch RCT was conducted between 2007 and 2009, and training of the surgical team including the anesthesiologist may have improved in recent years, resulting in reduced conversion rate. Interestingly, the type of hospital was related to the implementation of a laparoscopic approach. In 2015, all hospitals had implemented the LH, but implementation was faster in teaching hospitals compared to that in non-teaching hospitals. This is in line with the study of Pijnenborg and ter Haar who demonstrated the important contribution of residents in teaching hospitals in the implementation of LH in clinical practice [12]. We did not observe a relation between the age or gender of surgeon and the type of primary surgical approach in line with previous data [13, 14]. The safety of laparoscopy in the treatment of EC is established in eight RCTs that included mainly early-stage, low-grade EC [11]. There is strong evidence for the role of laparoscopy in the management of low-grade EC, yet for high-grade EC, data are still limited. In a recently published study, it was shown that LH and laparoscopic lymph node dissection were equally safe when compared to open procedures in high-grade EC [26]. Although numbers are relatively small, these data illustrate the shift of the indication towards the laparoscopic approach in high-grade EC treatment. This is supported by a follow-up date of the Gynecologic Oncology Group (GOG) LAP2 trial, which demonstrated that the outcome of patients with high-risk histology, including grade 3 endometrioid-type, serous, and clear cell carcinosarcoma, was not related to the type of surgical approach [27]. In our study cohort, only 10 EC patients underwent a LH with lymphadenectomy, since surgical staging was implemented from 2015 onward. Yet, since numbers of high-grade EC with laparoscopic surgery are limited, there is still a need to continue monitoring whether a laparoscopic approach can be extended to high-grade EC patients. This switch from open to laparoscopic surgery has great impact on the costs for healthcare. Even robotic-assisted laparoscopic hysterectomy was shown to be 17% cheaper when compared to AH, mainly due to a shorter hospital stay [28]. This benefit may be even more when conventional laparoscopic hysterectomy is performed and dependent on the use of expensive disposable supplies [29]. Whether advanced-stage EC can be treated by a minimal invasive procedure equally safe has not been studied so far.

This study has some limitations that need to be addressed. The surgical treatment of EC in the Netherlands is not centralized, and consequently, the current data reflect the clinical practice in one clinical oncology network in the Netherlands [23]. Since 2015, the surgical approach for EC is documented in the Netherlands Cancer Registry, demonstrating that 79% (66–83%) of the patients in 2015 with early-stage, low-grade EC were operated by a LH (data not shown). Based on our findings, we recommend to add the conversion rate and BMI to this Netherlands Cancer Registry database to monitor these in relation to annual cases in order to further improve the quality of care. Both the years of experience of individual surgeons with laparoscopic hysterectomy for benign indication and the experience of the surgical team have not been taken into account, while this may have influenced our data.

## Conclusions

In conclusion, LH has been well implemented in the surgical treatment of early-stage EC in a clinical oncology network in the Netherlands. Currently, 85% of the early-stage EC patients are operated by LH, mainly patients with low-grade tumor. Additional monitoring of conversion and complication rates might contribute to improved quality of care in the shift towards a laparoscopic approach for the treatment of EC.

## Abbreviations

AH: Abdominal hysterectomy
BMI: Body mass index
EC: Endometrial carcinoma/cancer
FIGO: International Federation of Gynecology and Obstetrics
LAVH: Laparoscopic-assisted vaginal hysterectomy
LH: Laparoscopic hysterectomy
TLH: Total laparoscopic hysterectomy
RCT: Randomized controlled trial
